# Complete Genome Sequences of the Type Strains Rouxiella badensis DSM 100043 and R. chamberiensis DSM 28324, Resolved Using Nanopore Long-Read Sequencing

**DOI:** 10.1128/mra.00156-23

**Published:** 2023-06-07

**Authors:** Saina Paul, Peter J. Anderson, Gregg J. Maynard, Mike Dyall-Smith, Timothy Kudinha

**Affiliations:** a School of Dentistry and Medical Sciences, Charles Sturt University, Orange, New South Wales, Australia; b Veterinary Biosciences, Melbourne Veterinary School, Faculty of Science, University of Melbourne, Parkville, Victoria, Australia; c NSW Health Pathology, Regional and Rural, Orange Base Hospital, Orange, New South Wales, Australia; University of Southern California

## Abstract

The complete genome sequences of Rouxiella badensis DSM 100043^T^ and Rouxiella chamberiensis DSM 28324^T^ were determined using Oxford Nanopore long-read sequencing and the Flye assembler. The former contains a circular chromosome of 4,964,479 bp and a circular plasmid of 116,582 bp; the latter contains a circular chromosome of 4,639,296 bp.

## ANNOUNCEMENT

The genus *Rouxiella* (family *Yersiniaceae*) was first described in 2015, with Rouxiella chamberiensis DSM 28324^T^ as the first species in the taxon (1). At the time of writing, the number of fully described species in this genus has increased to four, *R. chamberiensis*, R. aceris, R. silvae, and R. badensis; in nature, these species have been isolated from soils, the phyllosphere of strawberries, and tree sap, suggesting a close association with soils and plant ecosystems (2–7). Previous genome assemblies of the type strains *R. chamberiensis* DSM 28324 and *R. badensis* DSM 100043 contained 126 and 72 contigs, respectively (1, 2), making it difficult to perform useful bioinformatics analysis. To overcome this problem, we completed the genome assemblies of the type strains using Oxford Nanopore long-read DNA sequences. *R. chamberiensis* DSM 28324^T^ was found to have a single circular chromosome and *R. badensis* DSM 100043^T^ a single circular chromosome and plasmid, as determined using the Flye assembler (8) (see Table 1). Note that whole genomes produced solely from Nanopore long reads, such as these, will contain some frameshifted pseudogenes.

**TABLE 1:** Summary of DNA sequence assembly data

Feature	Data for strain:	
	*R. badensis* DSM 100043^T^	*R. chamberiensis* DSM 28324^T^
Chromosome size (bp)	4,964,479	4,639,296
GC content (%)	52.9	54.1
No. of tRNAs	75	78
No. of rRNAs	22	22
Read *N*_50_ (bp)	14,247	23,145
Mean read length (bp)	5,298	9,407
Coverage (×)	438	301
Total no. of genes	4,890	4,338
Genbank accession no.		
Chromosome	CP114060.1	CP114058.1
Plasmid	CP114059.1	No Plasmid

Rouxiella badensis DSM 100043^T^ and Rouxiella chamberiensis DSM 28324^T^ were purchased and shipped from the German Collection of Microorganisms and Cell Cultures (DSMZ). Stock cultures of each were prepared from single colonies grown on lysogeny broth (LB) agar and incubated at 28°C overnight. Frozen stocks were prepared by washing colonies from each plate with sterile LB medium containing 50% glycerol vol/vol and storing them at −80°C. For each genomic DNA extraction, the respective frozen stock culture was sampled and streaked onto potato dextrose agar (PDA), and a single colony was used to inoculate 2 mL of PD broth, which was incubated overnight at 28°C in a shaking incubator at 200 rpm.

Genomic DNA preparations were made using the Wizard genomic DNA purification kit (Promega, Australia), following the manufacturer’s instructions for isolation of genomic DNA from Gram-negative bacteria. DNA quality and quantity were assessed by UV spectrometry and agarose gel electrophoresis. DNA sequencing was completed at the Garvan Institute of Medical Research (Melbourne, Australia) using the Oxford Nanopore GridION platform with a FLO-MIN106 flow cell. The Oxford Nanopore Technologies SQK-LSK109 kit was used without shearing, generating a total of 426,035 reads for Rouxiella badensis DSM 10043^T^ and 151,485 reads for *R. chamberiensis* DSM 28324^T^. The reads were base called and quality controlled during sequencing using MinKNOW version 20.06.17 and Guppy version 4.0.11, respectively. Unless otherwise stated, all tools were run with default settings.

*De novo* assembly of the genome was completed using the Flye assembler version 1.2 (8) with Nanopore long-read DNA sequences running on the Geneious Prime version 2022.1.1 platform, with polishing set to 5 iterations (9). The chromosome and plasmid sequences of Rouxiella badensis DSM 100043^T^ were annotated using the NCBI Prokaryotic Genome Annotation Pipeline (PGAP) (10) and are predicted to contain a total of 4,890 genes, representing 3,765 protein-coding sequences, 22 rRNA genes, 75 tRNAs, and 9 noncoding RNAs (ncRNAs). Using NCBI PGAP, a total of 4,338 genes were predicted, encoding 3,528 protein-coding sequences, 106 RNA genes, which included 78 tRNA genes for Rouxiella chamberiensis DSM 28324^T^.

### Data availability.

The entire sequenced genome of Rouxiella badensis DSM 100043^T^ has been deposited at GenBank under the accession no. CP114060.1 and CP114059.1 (for its plasmid) and the BioSample accession no. SAMN32064794. The whole sequenced genome of Rouxiella chamberiensis DSM 28324^T^ has been deposited at GenBank under the accession no. CP114058.1 (11) and the BioSample accession no. SAMN32064795. The versions described in this paper for all chromosomes and plasmids are the first versions. The associated BioProject accession number for this project is PRJNA909035 (11). The raw data are available from the SRA via the accession no. SRX19145819 for *R. badensis* DSM 100043^T^ and SRX19145818 for *R. chamberiensis* DSM 28324^T^.
